# Systemic cell therapy for muscular dystrophies: The ultimate transplantable muscle progenitor cell and current challenges for clinical efficacy

**DOI:** 10.1007/s12015-020-10100-y

**Published:** 2020-12-21

**Authors:** C. Rosanne M. Ausems, Baziel G.M. van Engelen, Hans van Bokhoven, Derick G. Wansink

**Affiliations:** 1 Donders lnstitute for Brain Cognition and Behavior, Department of Human Genetics, Radboud University Medical Center, 6525 GA Nijmegen, The Netherlands; 2 Donders lnstitute for Brain Cognition and Behavior, Department of Neurology, Radboud University Medical Center, 6525 GA Nijmegen, The Netherlands; 3 Radboud Institute for Molecular Life Sciences, Department of Cell Biology, Radboud University Medical Center, 6525 GA Nijmegen, The Netherlands

**Keywords:** Cell therapy, Gene therapy, Mesoangioblast, Muscle regeneration, Muscle stem cell, Muscular dystrophy, Myogenic progenitor cell, Pericyte, Satellite cell, Skeletal muscle

## Abstract

The intrinsic regenerative capacity of skeletal muscle makes it an excellent target for cell therapy. However, the potential of muscle tissue to renew is typically exhausted and insufficient in muscular dystrophies (MDs), a large group of heterogeneous genetic disorders showing progressive loss of skeletal muscle fibers. Cell therapy for MDs has to rely on suppletion with donor cells with high myogenic regenerative capacity. Here, we provide an overview on stem cell lineages employed for strategies in MDs, with a focus on adult stem cells and progenitor cells resident in skeletal muscle. In the early days, the potential of myoblasts and satellite cells was explored, but after disappointing clinical results the field moved to other muscle progenitor cells, each with its own advantages and disadvantages. Most recently, mesoangioblasts and pericytes have been pursued for muscle cell therapy, leading to a handful of preclinical studies and a clinical trial. The current status of (pre)clinical work for the most common forms of MD illustrates the existing challenges and bottlenecks. Besides the intrinsic properties of transplantable cells, we discuss issues relating to cell expansion and cell viability after transplantation, optimal dosage, and route and timing of administration. Since MDs are genetic conditions, autologous cell therapy and gene therapy will need to go hand-in-hand, bringing in additional complications. Finally, we discuss determinants for optimization of future clinical trials for muscle cell therapy. Joined research efforts bring hope that effective therapies for MDs are on the horizon to fulfil the unmet clinical need in patients.

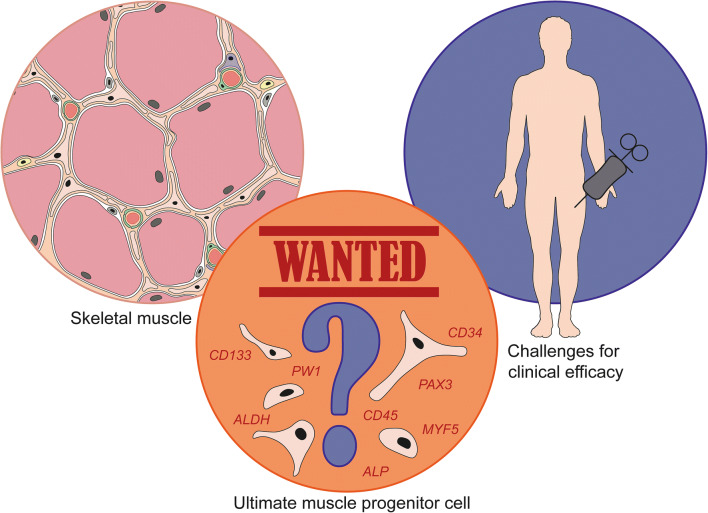

## Introduction

### Cell transplantation and skeletal muscle

Cell therapy, the administration of live cells in a patient for the treatment of a disease, was first successfully applied as a hematopoietic stem cell transplant (HSCT) in 1959 [1]. Cells from a patient with acute leukemia were destroyed by chemotherapy or radiation, after which cells from the immunologically matched donor, in this case the identical twin, were infused. These self-renewing cells found their way to the bone marrow, replicated and produced diverse blood cells. Dr. E. Donnall Thomas received the Nobel Prize in Physiology or Medicine in 1990 for the establishment of this successful treatment for leukemia and other blood conditions. Nowadays, many standardized transplantation protocols exist for various blood disorders [2].

Transplantation of skeletal muscle came into play in the early 1990s and aimed to restore dystrophin production in patients with Duchenne muscular dystrophy (DMD). Skeletal muscle seemed to be a suitable target for cell therapy, since muscle fibers mainly consist of postmitotic cells and there is limited cell turnover. Intercostal muscle, for example, shows an average turnover of 15.1 years [3, 4]. Whilst considerable progress in cell transplantation has been made in the past 30 years, a successful cell therapy supporting the regeneration of skeletal muscle in patients suffering from muscular dystrophies is still missing.

### Skeletal muscle formation and regeneration

**Fig. 1:**
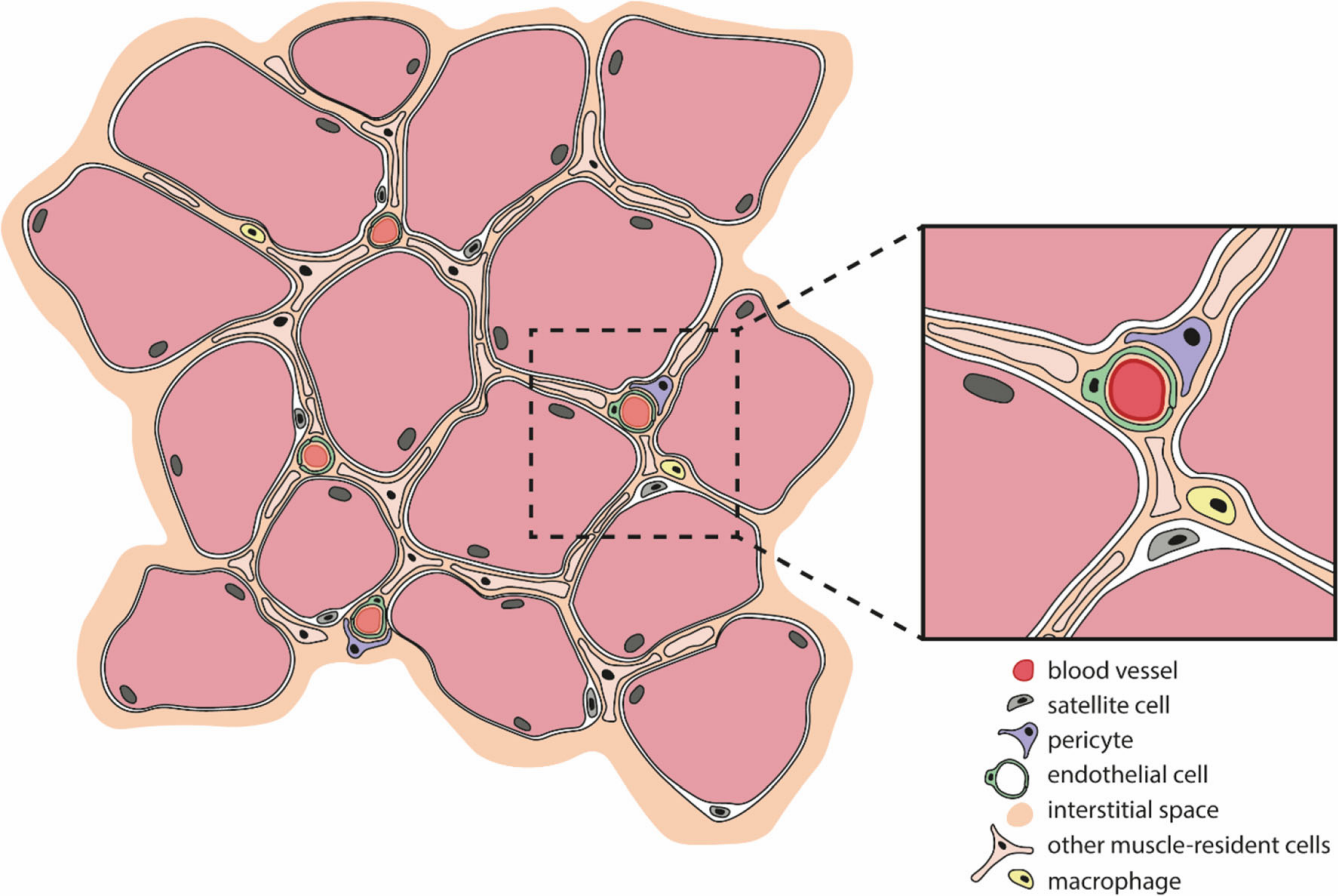
Skeletal muscle-resident cells. Schematic cross section of a healthy skeletal muscle bundle, containing more than a dozen individual muscle fibers (light red; nuclei at the periphery). Satellite cells (grey) are muscle-lineage committed progenitors that are located beneath the basal lamina of the muscle fibers, near the vasculature. In between the fibers are a variety of interstitial cells. Pericytes (purple) are one type and can be found wrapped around blood capillaries (insert). All these muscle-resident cell populations contribute to muscle repair and regeneration

Skeletal muscle formation, a process called myogenesis, occurs during embryonic development, and during postnatal muscle growth, regeneration and repair. Myoblasts, the early muscle cells with a single nucleus, proliferate and express muscle-specific genes leading to their fusion and the formation of multinucleated myotubes, which will ultimately form the mature myofibers [5]. Muscle maintenance is a continuous process depending on physical demand, injury and/or disease. Normal growth and replacement are mediated by a stem cell population termed satellite cells (SCs) (Fig. 1). These cells can be found beneath the basal lamina of muscle fibers and are stimulated after muscle injury to proliferate and differentiate to myoblasts, which then fuse to form new myotubes or fuse to existing myofibers, thereby contributing to muscle regeneration, which is further explained below [5, 6].

The disease state in several myopathies affects the functional capacity of SCs and consequently impedes muscle regeneration. Stimulating regeneration by the addition of myogenic cell types has therefore been the main goal of cell therapy for muscle diseases. This development started with the use of myoblasts and SCs, which led to disappointing clinical results due to limited survival and scarce migration of injected cells. Next, the field moved to other myogenic cell types, each with their own advantages and disadvantages. Most recently, mesoangioblasts (MABs) and pericytes have been pursued for muscle cell therapy. These myogenic progenitor cells possess various beneficial characteristics needed for an effective cell therapy approach, i.e. simple isolation, the ability to proliferate *in vitro*, systemic application, and the capacity to differentiate efficiently into skeletal muscle fibers *in vitro* and *in vivo*. Before we will discuss MABs and pericytes in more detail, we will review other myogenic progenitor cell types and cell populations that have been studied for their therapeutic potential over the last few years.

### Muscular dystrophies: variable clinical manifestations with a common high unmet medical need

Progressive wasting of skeletal muscle is characteristic for a subgroup of myopathies, the genetic and progressive conditions collectively called muscular dystrophies (MDs). There are over 30 (sub)types of MD with the most common diseases being myotonic dystrophy (DM), Duchenne muscular dystrophy (DMD), Becker muscular dystrophy (BMD), limb-girdle muscular dystrophy (LGMD), facioscapulohumeral muscular dystrophy (FSHD), oculopharyngeal muscular dystrophy (OPMD), distal muscular dystrophy (DD), Emery-Dreifuss muscular dystrophy (EDMD) and congenital muscular dystrophy (CMD) (summarized in Table 1). Since much can be learned from these MDs, we will discuss preclinical and clinical cell therapy work from the nine most prevalent forms (Tables 2 and 3). For a comprehensive overview of MDs, we refer to an excellent review by Shieh et al. [7].

**Table 1:** The nine most prevalent muscular dystrophies and their characteristics

Muscular dystrophy	Abbreviation	Mutation	Phenotype (focus on muscle)	Ref
		Single gene disorders		
*Becker muscular dystrophy*	BMD	*DMD* gene Preservation of the reading frame, synthesis of a truncated, but functional dystrophin.	Loss of ambulation and cardiac defects after the age 15, or asymptomatic far into adulthood. Compensatory transition to slow fiber type, as these are somewhat resistant to necrosis.	[182]
*Duchenne muscular dystrophy*	DMD	*DMD* gene Alteration of the open reading frame, loss of functional dystrophin. Disturbing the link between the cytoskeleton and the dystroglycan complex, causing membrane instability and fiber necrosis.	Affects most (proximal) limb muscles and axial muscles, but spares face muscles, including extraocular muscles (EOMs). Early loss of muscle fibers expressing MyHC-2X transcripts. Fast muscle fibers are mostly damaged, switch to slow type fibers.	[11, 183]
*Facioscapulohumeral muscular dystrophy*	FSHD1 (95%) FSHD2 (5%)	Derepression of the *DUX4* gene due to contraction of the D4Z4 repeat. Mutations in *SMCHD1, DNMT3B or LRIF1 gene*, encoding the protein controlling the methylation status of chromatin.	First signs mostly before the age of 20. Weakness is first and most detected in the facial muscles (but not EOMs), shoulder muscles and upper arms muscle, but weakness in other (axial) muscles also detected. Slow progression. Rarely affects the respiratory system (usually not the cardiac system), and most patients have an average life span. Especially type 2B fibers show a larger force deficit.	[11, 184]
*Myotonic dystrophy*	CDM, DM1 DM2	*DMPK* gene contains unstable (CTG)n repeat expansion. *CNBP* gene contains unstable (CCTG)n repeat expansion. Resulting in RNA toxicity associated with wide-spread abnormal alternative splicing.	DM1 is the most heterogeneous form also affecting other organs, with potential congenital or childhood-onset (CDM) and prominent CNS involvement. The primarily affected distal muscles in adult DM1 show mainly loss of type 1 fibers, whereas the predominantly affected proximal muscles in DM2 show mostly type 2 fiber atrophy.	[5]
*Oculopharyngeal muscular dystrophy*	OPMD	*PABPN1* gene contains expansion of alanine-encoding (GCN)n repeat. Leading to insoluble protein aggregates in the nuclei of skeletal muscle fibers.	Late-onset degenerative disorder. Most affected are EOMs (inducing ptosis), throat (causing dysphagia), and limbs (leading to proximal limb weakness). Muscle atrophy and fatty infiltration is suggested to be restricted to fast glycolytic fibers.	[185, 186]
		Multigene disorders		
*Congenital muscular dystrophy*	CMD	>13 genes are associated with CMD. Primary subtypes are caused by LAMA2 deficiency (MDC1A, mutations in *LAMA2*) or partial merosin deficiency (MDC1B, unknown gene), fukutin-related proteinopathy (MDC1C, mutations in *FKRP*), or acetylglucosaminyltransferase-like protein (LARGE)-related CMD (MDC1D, mutations in *LARGE*). Expansion of the spectrum by identification of various new genes encoding for both glycosyltransferases and structural proteins.	Early-onset, severe muscle diseases. Heterogenous phenotypes. Cardiac-, respiratory system and, in some subtypes, CNS and connective tissues are affected. Due to smaller type 1 muscle fibers a fiber size disproportion develops. Muscle wasting is caused by a combination of impaired developmental growth of type 1 fibers and hypertrophy of type 2 fibers.	[11, 187]
*Distal muscular dystrophy*	DD	The majority is genetically determined and 25 genes involved in diverse aspects of cell function have been identified. Mutations in proteins such as caveolin-3 (*CAV3*), dysferlin (*DYSF*), α-actin-1 (*ACTA1*), myotilin (*MYOT*), desmin (*DES*), and many others.	Variable phenotypes with an age-of-onset range from childhood to late adulthood. Initially very distal muscles affected, like the finger and toe extensor muscles. With disease progression, proximal muscles may become involved, but distal weakness remains the most severe.	[188]
*Emery-Dreifuss muscular dystrophy*	EDMD(1-4)	Mutations in *EMD*, *FHL1*, *LMNA* or other unknown genes that encode proteins in the nuclear envelope.	In the initial years, ankle and elbow contractures and spine rigidity appear. Later, the brachial and fibular muscle groups are affected. Induction of multiarticular contractures and induced cardiomyopathy.	[189]
*Limb-girdle muscular dystrophy*	LGMD	30 different subtypes. LGMD types are sarcoglycanopathy, calpainopathy, dysferlinopathy, and O-linked glycosylation defects (or dystroglycanopathy). Classification is based on genetic mutations and the inheritance pattern. LGMD type 1 consists of subtypes with autosomal dominant inheritance while type 2 includes forms of autosomal recessive inheritance. Calpainopathy, LGMD2A, is the most common form accounting for about 30% of cases and is caused by mutations in the *CAPN3* gene.	Variable age of onset. Mainly causing weakness of the proximal limb (the hip, shoulder, girdle) musculature. Many have associated cardiac findings. The bulbar muscle is often spared, although exceptions may occur.	[190]

*ACTA1;* α-actin-1, *CAV3;* caveolin-3, *DES;* desmin, *DYSF;* dysferlin, *EMD;* emerin, EOMs; extraocular muscles, *PABPN1;* poly-adenylate (poly(A)) binding protein nuclear 1, *DMPK;* dystrophia myotonica protein kinase, *CNBP/ZNF9;* cellular nucleic acid binding protein/zinc finger protein 9, *SMCHD1;* structural maintenance of chromosomes flexible hinge domain containing 1, *LAMA2;* laminin alpha-2, MDC1A/B/C/D; muscular dystrophy congenital type 1a/b/c/d, *FKRP;* fukutin related protein, *LARGE1;* LARGE xylosyl- and glucuronyltransferase 1, *MYOT* myotilin

**Table 2:** Pre-clinical studies using various muscle progenitor cell types in cell therapy approaches for muscular dystrophies

Cell type	Abbreviation	Administration	(Pre-)clinical study	Comments/ pitfalls
Aldehyde dehydrogenase 1A1 cell	ALDH cell	i.m.	Only the CD34^-^ fraction of human ALDH^+^ cells was myogenic after transplantation in de TA of immunodeficient *scid* mice [53].	Unclear if systemic delivery is possible, if so, the high proliferative capacity of ALDH cells is positive.
CD133^+^ (muscle derived) progenitor cell	CD133^+^ cell	i.m. and i.a.	Genetically corrected CD133^+^ cells obtained from DMD patients produced dystrophin and recovered muscle morphology and function in immunodeficient *mdx* mice [55]. Intra-arterially injected autologous engineered canine CD133+ cells restore dystrophin expression in GRMD dogs improving clinical outcome [58].	CD133^+^ cells are a heterogenous population. Specific subpopulations were used. CD34 to decipher between activated (CD34^+^) cells and more quiescent (CD34^−^) cells. CD56, a marker of muscle progenitors, influences the regenerative capacity [57].
Mesenchymal(-like) stem cell	MSC	i.v. and i.m.	MSCs restored cytoplasmic expression of dystrophin, reduced central nucleation, and rescued the expression of mouse mechano growth factor in immunosuppressed *mdx* mice [63].	MSCs can secrete trophic factors that can influence endogenous mechanisms of tissue regeneration [61]. Anti-inflammatory activity may exert additional positive effects [64].
Mesoangioblast	MAB	i.a.	After a single i.a. injection, SG expression was found in >90% of muscle fibers in the TA muscle of α-SG-null mice. Protein expression was restored to roughly 60% of wild-type levels [122].	Delivery was optimized as MABs were exposed to combined pretreatment with SDF-1 or TNFα and expression of α4 integrin [122].
Muscle-derived stem cell including -Muscle stem cell -Side population cell	MDSC - MuStem cell - SP cell	i.a. and i.m.	MDSCs from normal dog muscle restored some dystrophin expression in myofibers of GRMD dogs, after i.m. or i.a. injection [66]. Murine SP cells exhibited the potential to give rise to both myocytes and SCs after i.m. transplantation into immunodeficient SCID/bg or NOD/scid mice [68, 70].	Heterogenous group of muscle SP cells show low abundance and absence of specific SP cell markers [69]. Further characterization is needed before MDSCs should be considered for therapeutic approaches. The myogenicity of SP cells depends, in some articles, on the presence of myoblasts and/or specific culture conditions [68, 69]. SP cells isolated from dystrophic muscle differentiate along fibro-adipogenic lineage [69].
Myoendothelial cell		i.m.	Human myoendothelial cells injected into immunodeficient *scid* mice regenerate myofibers. They do so more efficiently than CD56^+^ myogenic progenitors, which could be partly explained by their faster proliferation rate and higher resistance to oxidative stress, as shown *in vitro* [72]. Another group used the same name for cells isolated from the mouse endomysium that were able of differentiating into muscle and endothelial cells after transplantation in *NOD/shi-scid* mice [75].	Human myoendothelial cells did not form hybrid myofibers, but only form *de novo* fibers [72]. The cell population used could partly consist of SP cells [75].
Pericyte	PC	i.m. or i.a.	GRMD dogs were treated with local or systemic injections of pericytes together with different immunosuppression regimes with steroids. Variable dystrophin expression was observed from different biopsy samples (10%– 70%) for all dogs however, a significant increase in force production in the treated leg was seen [128].	Donor wild-type cells significantly ameliorate symptoms of canine DMD, whereas autologous genetically corrected cells were less effective [128].
PW1^+^/Pax7^-^ interstitial cell	PIC	i.m.	PICS isolated from mouse or porcine muscles are myogenic *in vitro* and can contribute to skeletal muscle regeneration *in vivo* [76, 77].	Enhanced skeletal muscle repair was not caused by a direct fusion of pPICs, since these were eliminated by the host immune system, but rather due to the stimulation of the endogenous stem pool [77].

i.m. intramuscular, i.v. intravenous, i.a. intra-arterial, TA tibialis anterior

**Table 3:** Clinical studies concerning cell therapy approaches for muscular dystrophies, distinct from the use of myoblasts and satellite cells

Type of study	Cell type	Administration	Number patients	Cell number	Effects
Double-blind phase I clinical trial Torrente et al. 2007	CD133^+^	i.m.	8 Stem cell group *n* = 5 Sham group *n* = 3	Three parallel injections of 2x10^4^ cells at 1 mm interdistance.	Autologous transplantation of CD133^+^ cells in three injection trajectories in the abductor digiti minimi muscle of eight DMD patients showed no side effects, an increase in capillary vascularization, no effective integration in muscle fibers [59].
Non-randomized open-label phase I–IIa clincal trial Cossu et al. 2015	Pericyte	i.a.	5	Doses based on kg/body weight and in multiple limb arteries. Each limb received similar doses in of cells in respect to its mass. The exact injected doses are reported in Appendix Table S4 of the original article.	In five Duchenne patients escalating doses of donor-derived cells were administered, 4 times at two-month intervals, in limb arteries under immunosuppressive therapy. Clinical, laboratory and MRI analysis revealed that the study was relatively safe [129]. The effects on muscle function were inconclusive. Stabilization but no functional improvement was observed in 2 out of 3 ambulant patients. However, MRI showed disease progression in 4 of 5 patients [129].

Developmental delay, shown by a reduced number of myofibers at birth, is typically present in newborns with a congenital form of MD, while adult onset MDs are characterized by progressive wasting of initially correctly formed skeletal muscle tissue [8]. Being highly variable in terms of age of onset, severity of symptoms, clinical patterns and genetics, the MDs are troublesome to diagnose, challenging to treat and, so far, impossible to cure. Their total combined prevalence ranges between 19.8 and 25.1 per 100.000 person-years, but can vary greatly amongst geographical regions [9]. Note that different muscles, and consequently fiber types and SCs, may show a difference in susceptibility to muscular atrophy [10, 11] (Table 1). These properties will likely also influence the response to cell therapy.

Causative mutations for MDs often affect genes that encode proteins of the dystrophin-associated glycoprotein complex, but mutations in other genes have been shown to participate in the pathogenesis as well. Most of these mutations, directly or indirectly, affect proteins that localize at the sarcomere and Z band, or are nuclear membrane components [12–14]. Changes in all these structural proteins generally lead to the loss of muscle cell integrity and damage to the fibers. In the end, this myopathic process compromises mobility and can lead to respiratory distress, heart failure and premature death.

## Wanted: the ultimate transplantable muscle progenitor cell

The present status of muscle cell therapy for the nine most common forms of MD clearly illustrates the challenges and bottlenecks that need to be solved in the establishment of safe and effective treatment. Currently, one of the major difficulties is the choice of a suitable, transplantable cell type. Research using myoblasts and SCs has been extensively reviewed elsewhere [15–17]. From the time when these reviews were published clinical progress with myoblasts and SCs has been limited [16, 18, 19]. Importantly, as SCs and their myoblast progeny lack the ability to cross the muscle endothelium, they must be injected intramuscularly at close intervals (every ~2 mm^3^) and are therefore unable to systemically treat MDs [15, 18, 20]. Although this procedure might still be of some benefit to certain MD patients, the fact that these cells can only be used to treat individual muscles moved the main focus to other muscle progenitor cells. These progenitors hold self-renewal capacity and are resident in the muscle, the endomysium or are associated with the vasculature. In addition, we also discuss the potential of induced pluripotent stem cells (iPSCs). The donor cell source likely affects differentiation efficiency. When skeletal muscle is chosen as source for adult stem cells, these muscle-derived cells show a preference for differentiation into the myogenic lineage [21–23]. The same holds for the generation of iPSCs, which tend to have a durable epigenetic memory from the cell used for reprogramming that affects their myogenic potential [24, 25].

It is important to note that the generation of a comprehensive overview of the different progenitor muscle cells reported in the literature appeared complicated by the lack of accurate descriptions of these cells. We found that many papers are incomplete or make use of heterogeneous populations of progenitor cells, with overlapping molecular makers, anatomical localization and different methods of isolation [26].

### Myoblasts and satellite cells: how the mighty have fallen?

In 1989, the groups of Louis Kunkel and Terence Partridge pioneered muscle cell transplantation in MD models [27, 28]. Allogenic neonatal mouse myoblasts were the first cells to be intramuscularly transplanted into a dystrophin-deficient DMD model with the goal to form new dystrophin-positive fibers. Although encouraging, the results in mice could not be recapitulated in patients, which were injected with muscle stem cells harvested from healthy human immunocompatible donors [18, 29–32]. Only low expression percentages of normal dystrophin were detected posttransplantation in muscle biopsies [29–32] and, with the exception of one study [33], no functional muscle improvement in the transplanted limb was measured. Functional effects in these studies were limited by cell death after transplantation and immune rejection, together resulting in a limited number of cells that survived *in vivo*, and a scarce migration of injected cells. Recently, in an attempt to increase engraftment, limit rejection and restore dystrophin expression, fused myoblasts from healthy donors and DMD-affected donors were i.m. injected into *mdx/scid* mice [34].

SCs are able to maintain their stemness due to their self-renewal capacity, implying that SCs are preferred over myoblasts for a successful therapy. They are considered to be the *bona fide* stem cell of skeletal muscle. SCs are positioned between the basal lamina and the sarcolemma of muscle fibers (Fig. 1) [6]. In this niche, quiescent SCs can respond to damage or disease due to the local release of cytokines, growth factors or cell differentiation factors like NOTCH and WNT. The factors are secreted by muscle tissue itself and nearby macrophages or fibroblasts. High expression of *NUMB*, an antagonist of NOTCH signaling, leads cells to go back in quiescence [5, 35]. Quiescent SCs express *PAX7*, *MYF5*, *CD34* and generally also *PAX*3 [36, 37]. SCs in which p38α/β MAPK is asymmetrically activated, undergo myogenic commitment [5, 38]. A group of highly conserved myogenic regulatory factors (MRFs) is responsible for the entrance in the cell cycle and further myogenic differentiation. Activated SCs loose *CD34* expression and instead start expressing *MYOD*, a well-known member of the MRF family. Co-expression with myogenin (*MYOG*) activates terminal differentiation of the muscle cells [37]. The cascade responsible for muscle differentiation is additionally influenced by a multitude of cytokines, circulating hormones and exosome-secreted signals [5, 37]. This process of skeletal muscle formation, growth and maintenance in healthy subjects or patients with MD is elaborately discussed elsewhere [5, 26, 39].

The therapeutic value of SCs has been examined in several transplantation studies [40, 41]. However, many of the same practical limitations and safety concerns seen for myoblasts appear to restrict the use of human SCs in the clinic. The low proliferative capacity *in vivo*, limited migration through muscle tissue [42], in addition to the immune reactions against donor cells [43, 44], were highlighted as the main concerns (reviewed in [15, 45]). Trying to overcome these challenges, researchers delivered a large number of allogeneic myogenic cells under immunosuppressive conditions using multiple injections [15, 46]. However, such high-density injection protocols seem to be feasible only for dystrophies where small muscles are locally affected, e.g. in OPMD [19, 47]. Accordingly, the search for a cell type more suitable for a systemic muscle cell therapy continued.

### A diverse repertoire of muscle progenitor cells

Since the 1990s the repertoire of cells used for cell therapy has expanded tremendously. There is a myriad of cells reported to participate in myofiber regeneration under experimental conditions. Some of these stem cells or myogenic progenitors have an additional beneficial characteristic: they can be administered systemically. While intramuscular (i.m.) injections were used for myoblasts and SCs, for a wider distribution systemic delivery of cells via the vasculature is clearly advantageous to access the entire muscular system. The predominant route used is intravenous (i.v.) cell injection, which is associated however with considerable cell loss due to sequestration in the lungs as a consequence of the pulmonary first-pass effect [48]. More demanding, intra-arterial application (i.a.) is feasible and avoids capillary filters as liver and lung. Distribution needs to be closely monitored as cells can be entrapped in other organs including the brain [48–51].

#### Aldehyde dehydrogenase 1A1 (ALDH^+^) cells

Aldehyde dehydrogenase 1A1 (ALDH) is a marker of bone marrow, umbilical cord blood and peripheral blood primitive progenitors. In addition, ALDH activity is used to identify stem cells. ALDH^+^ cells can be isolated from skeletal muscle for cell therapy. In DMD patients, their presence is even increased [52]. In one publication, after i.m. injection into a mouse model, ALDH^+^ and CD34^–^ cells isolated from human skeletal muscles, proliferated, robustly contributed to muscle regeneration and even contributed to the pool of SCs [53].

#### CD133^+^ cells

Blood and skeletal muscle-derived CD133^+^ cells are defined by their expression of CD133, a surface marker of various stem and progenitor cells [54]. The cells possess myogenic capacity as they contribute to muscle regeneration and are able to colonize the SC niche, while restoring dystrophin expression in dystrophic severe combined immunodeficiency s*cid*/*mdx* mice [55, 56].

When compared to *bona fide* SCs, human muscle-derived CD133^+^ cells displayed a superior regenerative capacity after injection in immunodeficient mice [57] (Table 3). The increased presence of human cells in a SC position, an elevated expression of human proteins in fibers and a better dispersion of CD133^+^/CD34^+^ cells in the host muscle showed that CD133^+^ cells outperformed human SCs on multiple aspects [57].

The positive results obtained by CD133^+^ cell injections into murine models of DMD was followed by a translational study using CD133^+^ cells isolated from dystrophic dog muscle. After i.a. injection, autologous and engineered canine CD133^+^ cells restored dystrophin expression in the golden retriever muscular dystrophy (GRMD) dog model. Moreover, an improvement in the clinical outcome measures, and, in many cases, a preservation of walking ability within the first year of treatment was seen. Of note, while trying to boost dystrophin expression with an extra cell infusion, an immune response was triggered which significantly worsened the clinical condition in three out of five treated GRMD dogs [58].

In a double-blind phase I clinical trial, transplantation of autologous non-edited CD133^+^ cells showed no side effects, but neither was there effective integration of transplanted cells into muscle fibers seven months after injection [59]. Of the five DMD patients treated with these stem cells, four patients had an increased number of capillaries per muscle fiber and two of them had an unexplained switch towards fast myosin myofibers.

When considering CD133^+^ cells for clinical application, allogenic cells might be the preferred choice for the treatment of DMD, since CD133^+^ cells derived from DMD muscle showed lower overall performance after i.m. injection into an immunodeficient, non-dystrophic, mouse muscle [60]. DMD derived CD133^+^ cells did not form SCs and produced significantly fewer muscle fibers. This may be due to a chronic, dystrophy-related transformation within DMD tissues leading to upregulation of CD133 expression in non-myogenic cells within the muscle [60].

#### Mesenchymal(-like) stem cells (MSCs)

Mesenchymal stem cells (MSCs) possess multilineage differentiation capacity and were formerly isolated from adult and fetal bone marrow. Nowadays, MSCs can be isolated from various tissues, including skeletal muscle [61, 62]. The potential of MSCs has been demonstrated in several animal models, for example by cells obtained from adult human synovial membrane. I.m. transplantation of MSCs into immunosuppressed *mdx* mice restored cytoplasmic expression of dystrophin and reduced central nucleation. Moreover, expression of mouse mechano growth factor, an important factor that influences local muscle maintenance, was partially rescued [63]. Although cells were found in diverse tissues, including the lungs, systemically injected MSCs were shown to respond to local signals, as preferred homing and myogenic differentiation was only seen within skeletal muscle [63].

Next to direct differentiation at the dystrophic target tissue, MSCs exert secondary therapeutic effects via the production of paracrine factors. These factors inhibit apoptosis, stimulate endogenous cell proliferation, and/or activate tissue-resident stem cells at the site of injury. For the treatment of dystrophic phenotypes, it is of interest that MSCs possess anti-inflammatory activity [61, 64]. DMD patients have a prolonged inflammatory milieu, as a result of prolonged muscular strain, which appears to enhance muscular atrophy. Although dystrophin production is needed to reverse the phenotype, it is clear that inflammation, nowadays treated by corticosteroids, also has an impact on disease progression in DMD [64].

#### Muscle-derived stem cells (MDSCs)

Muscle-derived stem cell (MDSC) is an umbrella term for multipotent cells obtained from muscle via different routes [65]. It remains difficult to precisely discriminate, compare and name these cells as a result of their high variability after purification methods. Two subtypes are discussed here: muscle stem cells and side population cells.

##### Muscle stem cells (MuStem cells)

Rouger et al. [66] explored the transplantation efficiency of MDSCs, which they alluded to as muscle stem cells (MuStem cells), isolated from healthy dog muscles. When transplanted into the bloodstream of immunosuppressed GRMD dogs, the cells showed efficient homing as they reached the hindleg skeletal muscle, which led to local dystrophin expression and SC replenishment. Importantly, systemic delivery of MuStem cells led to prolonged dystrophin expression with increased myofiber regeneration and an enduring stabilization of the clinical status of the treated dogs [66]. The human counterpart of the canine MuStem cell could be identified and also displayed skeletal muscle regeneration after i.m. delivery into immunodeficient host mice, suggesting that human MuStem cells could be a suitable candidate for cell therapy [67].

##### Side population cells (SP)

Side population (SP) cells are MDSCs defined by expression of the hematopoietic stem cell marker SCA-1, no expression of any additional SC marker and exclusion of Hoechst dye [68]. These SP cells exhibit the potential to give rise to both adult myoblasts and SCs after i.m. transplantation. Several research groups studied the potential of SP cells, but due to differences in the characterization of the cells and therefore variation in cell subpopulations, some reports seem to contradict each other. Asakura *et al.* revealed that *in vitro* both subfractions of SP cells, CD45^−^ and CD45^+^ cells, exhibited myogenic potential [68], albeit that myogenesis was only seen after co-culture with primary myoblasts. In contrast, SP cells isolated by Penton *et al.* did not require co-culture with myogenic cells. These SP cells were negative for CD45 and the vascular marker CD31, but did express PAX7, SCA1 and the mesenchymal progenitor marker PDGFRα [69].

When isolated from cardiotoxin-injured muscle or dystrophic mice models, SP cells lost myogenic potential and instead differentiated along fibro-adipogenic lineages [69]. Muscle damage seems to affect the lineage choices of muscle SP cells, possibly unraveling a role for muscle SP cells in the fibrotic process in MDs. Even though the CD31^−^ CD45^−^ subpopulation of SP cells is the smallest fraction among the three CD31/CD45 SP cell populations in healthy muscle, this subfraction displayed the highest myogenic potential both *in vitro*, with co-culture of myoblasts, and *in vivo*. I.m. injection of CD31^−^ CD45^−^ SP cells into injured skeletal muscle urged active proliferation and myofiber generation [70]. In a follow-up study, co-injection of green fluorescent protein (GFP)-positive myoblasts with CD31^−^/CD45^−^ SP cells suggested an indirect but important supportive role for SP cells. When transplanted into the tibialis anterior of immunodeficient *NOD/scid* mice or dystrophin-deficient *mdx* mice, CD31^−^/CD45^−^ SP cells stimulated myoblast proliferation and migration, leading to more fiber generation and a broader distribution [71].

Combining all these observations, it seems entirely possible that the muscle SP cell fraction contains multiple types of progenitor cells, each with a restricted potential and possible dependence on additional myogenic cells. It therefore still remains to be clarified which subpopulation of SP cells contains most myogenic potential and which subfraction mainly gives rise to adipocytes and osteocytes [68, 69].

#### Myoendothelial cells

Myoendothelial cells co-express the three endothelial and myogenic markers CD34, CD56 and CD144, and can contribute to postnatal muscle growth [72]. Myogenic and endothelial cells derive from a common precursor since both cell types originate from somites. The discovery that myoendothelial cells produce early myogenic stem cells with the ability to replenish the SC population suggests the presence of a certain hierarchy within the development and regeneration of human adult skeletal muscle [73–75].

The transplantation of human myoendothelial cells in *scid* mice surprisingly outperformed the regenerative potential of myogenic CD34^−^/CD56^+^/CD144^−^ cells or endothelial CD34^+^/CD56^−^/CD144^+^ cells [72]. This may be a consequence of their increased proliferation rate and greater resistance to oxidative stress [22].

After transplantation into mice, myoendothelial cells were capable of differentiating into skeletal muscle and vascular endothelial cells [75]. Cells were first sorted on CD34^+^, a myogenic cell marker, and secondly on CD45^−^, considered to be a hematopoietic cell marker. Only the CD34^−^ population showed myogenic markers after sorting, however only the CD34^+^/CD45^−^ cell pool showed myogenic potential *in vivo*. Six weeks after cell transplantation, the engrafted GFP-positive cells were detected in the tibialis anterior muscles of three of the five injected mice, but in none of the CD34^−^/45^−^ injected mice. The transplanted CD34^+^ cells fully differentiated into skeletal muscle and vascular endothelial cells *in vivo*.

Tamaki et al. provide a good example of the challenge of sorting a homogenous cell population and investigating the lineage potential thereof. Even though further characterization excluded hematopoietic and endothelial cells from the cell transplantation pool, muscle SP cells might still be present in the CD34^−^/45^−^ and CD34^+^/45^−^ fractions, since the muscle SP cells are a mixture of CD34^+^ and CD34^−^ cells [75].

#### PW1^+^ interstitial cells (PICs)

PW1^+^ interstitial cells (PICs) are a population of muscle-resident stem cells, that are located in the interstitium and express the cell stress mediator PW1/PEG3 (paternally expressed 3), but do not express other muscle stem cell markers such as *PAX7* [76]. PICs are myogenic *in vitro* and *in vivo* and display myogenic potential comparable to freshly isolated SCs. In addition, PICs efficiently self-renew, giving rise to more PICs in addition to SCs and myofibers [76].

By using a preclinical porcine skeletal muscle injury model, it was shown that allogeneic porcine PICs significantly enhanced myofiber regeneration and neocapillarization after i.m. injection. This effect was probably due to stimulation of the endogenous stem cell pool, since PICs express and secrete a multitude of pro-regenerative growth factors and cytokines. The direct contribution of the PICS to myofiber regeneration and neocapillarization was negligible [77].

## Mesoangioblasts and pericytes

Mesoangioblasts (MABs) and pericytes, two cell types isolated from embryonic and postnatal tissues respectively, appeared to possess critical properties needed for a successful cell therapy approach. A clinical trial with this cell type was performed in 2015 [78] (Table 3). Due to the high potential of MABs and pericytes, we have devoted a separate section to these cells.

### The microvasculature as niche and angiogenesis as distribution route

MABs are mesenchymal-like cells associated with the wall of the aorta. These myogenic cells are thus from non-somite, vascular origin [63, 74, 75, 79–84]. MABs use the microvasculature and angiogenesis events as progenitor cell niche and distribution route, respectively, during which progenitors are generated and spread throughout various postnatal tissues [20, 85]. MABs leave the blood vessels and enter the surrounding mesenchyme where they are exposed to local signals, allowing them to integrate and fully differentiate into mesodermal tissues such as blood, cartilage, bone and smooth, skeletal and cardiac muscle [73]. The idea that MABs, isolated from explant cultures of embryonic dorsal aorta, are blood borne is supported by the presence of known vascular endothelial markers such as VE-cadherin, β3-integrin and P-selectin, in addition to myogenic markers (MYOD, MYF5, DESMIN, MNF, c-MET and M-cadherin) [74].

### Myogenic fate regulation of MABs is *Pax3*-dependent

The muscular fate determination of myogenic progenitors is influenced by the paired box/homeodomain transcription factors of the *Pax* family. Mouse SCs express *Pax3* and *Pax7* and once they become myoblasts, *Pax7* expression goes down, while *MyoD* and *Myf5* expression remain. Surprisingly, *Pax7* expression is not found in MABs. The skeletal muscle fate of these cells is only *Pax3*-dependent [86, 87]. During embryonic development, skeletal muscle is formed from the dorsal somite, the dermomyotome, *Pax3* is expressed in the multipotent cells of this structure [88]. *Pax3* is not only essential for the survival of cells in the dermomyotome, but also plays an important role in the migration of these cells outside the somite [86]. When outside, in the limb buds for example, the myogenic determination gene Myf5 is activated [89]. Genetic labeling showed that vessel-derived progenitor cells and cells of the myotome both originate from *Pax3*^+^ multipotent cells of the paraxial mesoderm [90].

### Pericyte, the postnatal equivalent of MAB

Interestingly, tracing *Pax3*^+^ GFP-labeled MABs also revealed *Pax3*^+^ cells in postnatal blood vessels. Although their frequency appears to be lower than the embryonic correlate, these *Pax3*^+^ progenitors, called pericytes, are considered the descendants of the multi-potent *Pax3*^+^ cells present during embryogenesis [86, 88, 90–92]. The cells delivered through fetal angiogenesis remain dormant within steady-state tissues and can be activated during postnatal events.

Pericytes are contractile cells in close proximity with the endothelial cells of small blood vessels [93]. They can be found wrapped around blood capillaries, precapillary arterioles, postcapillary venules, and collecting venules (Fig. 1) [94], where they regulate capillary barriers, endothelial proliferation and capillary diameter by integrating and coordinating neighboring endothelial cell response [92]. In contrast to MABs, pericytes have no unlimited self-renewal capacity *in vitro*. They adopt a large, flat morphology after approximately 20-30 population doublings and undergo senescence [20, 91]. Unlike MABs, pericytes spontaneously differentiate into skeletal muscle myotubes in low-serum culture conditions [95]. Skeletal muscle tissue appears to be the most effective source for pericytes with myogenic potential [22].

### Establishing a comprehensive pericyte identity

It is difficult to discriminate pericytes from other interstitial progenitor cells. Pericyte abundance, morphology and marker identity differ throughout the body [96–98]. Moreover, there is lack of a specific pericyte maker, which makes it challenging to exactly characterize these progenitor cells. In contrast to MABs, pericytes lack endothelial markers CD31, CD34, and KDR. Markers such as M-cadherin, N-CAM, cytokeratins or neurofilaments (with the exception of nestin) are also not present. Neither are myogenic markers, MYOD, MYF5 and MYOG, apart from PAX3 [20]. The difference in marker expression between muscle progenitors may be correlated with the different localization of the cell types. SCs are located next to the basal lamina of muscle fibers, while MABs occupy an endothelial position. Pericytes, on the other hand, are located underneath the basal lamina of the small vessel, where they are completely embedded within the endothelial cell basement membrane.

Markers that have been used to identify pericytes include smooth muscle α-actin (αSMA) [99], desmin (DES) [100], high molecular weight melanoma antigen (HMW-MMA) (called NG2 in the mouse) [101, 102], platelet-derived growth factor receptor (PDGFR)-β [103], aminopeptidase A and N [104, 105], the regulator of G-protein signaling-5 (RGS5) [106, 107], and the promoter trap transgene *XLACZ4* [108] (Table 4). The subfraction of pericytes with skeletal myogenic potential can be recognized by the absence of PDGFRα and the presence of alkaline phosphatase (ALP) in murine and human tissues [20, 91, 95, 109, 110]. Moreover, expression of PW1/PEG3 is related to progenitor cell competence, since silencing *PW1*/*Peg3* leads to MYOD degradation and consequently inhibits myogenic potential *in vitro*. Additionally, without PW1/PEG3 cells are unable to modulate the junctional adhesion molecule-A (JAM-A) and are therefore incapable of migrating across the vessel wall and engrafting into damaged myofibers [111]. It is good to keep in mind that all markers nowadays used for selection are dynamic in their expression. They may be up- or down-regulated between organs, in conjunction with developmental phases, pericyte maturity [112, 113], diverse pathological states and *in vitro* culturing [114, 115].

**Table 4:** Commonly used pericyte markers

Marker	Gene symbol	Description	Example of other cell types expressing the marker	Ref
Alpha-smooth muscle actin (αSMA)	*ACTA2*	Cytoskeletal contractile protein; quiescent pericytes do not express αSMA; expression in pericytes is commonly upregulated in tumors and during inflammation.	Smooth muscle, myofibroblasts, myoepithelium.	[20, 95, 99, 191]
Aminopeptidase N (AP-N) or CD13 and Aminopeptidase A (AP-A; alanyl membrane aminopeptidase)	*ANPEP*	Membrane zinc-dependent metalloprotease; expression increased in vasculature of tumors and wound healing tissue as compared with normal resting tissues.	vSMCs, inflamed and tumor endothelium, myeloid cells, epithelial cells in the kidney, gut; useful marker for brain pericytes.	[104, 105, 192]
Alkaline Phosphatase (ALP) or Tissue-Nonspecific ALP (TN-ALP)	*ALPL*	Membrane-bound glycosylated enzyme; plays a role in bone mineralization. Cell membranes of many cell types have ALP activity, however in skeletal muscle only pericytes and endothelial cells express ALP.	Undifferentiated pluripotent stem cells, cancer cells and osteoblasts have elevated levels.	[20, 91, 95, 109]
Desmin (DES)	*DESMIN*	Intermediate filament protein; predominantly expressed in muscle cells.	Skeletal, cardiac, smooth muscle. Useful pericyte marker outside skeletal muscle and heart.	[20, 100]
Chondroitin sulfate proteoglycan 4 (CSPG4) or Neuron-glial antigen 2 (NG2)	*HMW-MMA* or *NG2* in mice	Chondroitin sulfate proteoglycan; involved in cell survival, migration and angiogenesis; expression differs e.g., only arteriolar, but not venular pericytes are positive for NG2.	Developing cartilage, adipocytes, vSMCs, neuronal progenitors, oligodendrocyte progenitors, mesenchymal stem cells, osteoblasts, melanocytes, smooth muscle cells and macrophages	[20, 101, 102, 193]
Platelet-derived growth factor receptor-beta	*PDGFR-β*	Receptor tyrosine kinase; plays a role in pericyte recruitment during angiogenesis; useful marker for brain pericytes.	Interstitial mesenchymal cells during development; smooth muscle; in the CNS certain neurons and neuronal progenitors; myofibroblasts; mesenchymal stem cells.	[20, 95, 103]
Paternally Expressed 3 (PEG3) or PW1/PEG3	*PEG3*	Zinc finger protein; involved in cell proliferation and p53-mediated apoptosis; involved in pericyte migration across the vessel wall.	Expressed in various progenitor/stem cells in all adult tissues, including the intestine, blood, testis, CNS, bone, skeletal muscle, and skin.	[194]
RGS5 (regulator of G protein signaling 5) Rgs5	*RGS5*	GTPase-activating protein.	Heart (cardiomyocytes), lung, skeletal muscle and small intestine (vSMCs), and at lower levels in brain, placenta, liver colon, and leukocyte.	[106, 107, 195]

### Myogenic fate regulation of pericytes begins at the onset of differentiation

When investigating the differentiation process, it was found that mouse pericytes never expressed *Pax7*, *Myf5* nor *MyoD* during proliferation, but activated these genes at the final stages of differentiation. This activation was concomitant with that of myogenin and just before the expression of myosin heavy chain proteins in myotubes. The temporal expression pattern is different in SCs, which do express *Pax7*, *Myf5* or *MyoD* during proliferation and activate myogenin before myosin heavy chain [20].

ALP^+^ pericyte-mediated myogenesis seems modest in adult muscle as the ALP^+^ cells mainly contribute to skeletal muscle during the first few weeks of postnatal growth. The process of ALP^+^ cell contribution becomes infrequent in adult mice [109]. However, pericytes can respond to postnatal repair events. Vascular ALP^+^ pericyte progenitors mainly contributed to the growth of muscle fibers by entering the SC niche, albeit direct fusion with developing muscle fibers occurred as well. The ALP^+^ pericyte-derived SCs could self-renew and maintained a steady contribution to the SC pool [109].

Acute skeletal muscle regeneration and chronic skeletal muscle regeneration showed increased ALP^+^ cell counts at these sites, respectively five and three times more ALP^+^ cells compared to controls. Most studies indeed found an increase of pericytes in in myopathic muscle biopsies, especially when regeneration is present [116]. However, some studies discovered a reduced number of pericytes in specific forms of MD [117].

### The role of trophic factors in myogenic fate regulation by pericytes

The functional benefit of stem cell therapies relies on the *in situ* differentiation of the grafted cells. Besides intrinsic cellular properties, differentiation potential is dependent on trophic factors. Paracrine factors secreted by pericytes may act on adjacent muscle stem cells and exert regulation of their postnatal fate. An ELISA for 121 cytokines and growth factors on pericyte culture supernatant showed that it contained two factors for receptors highly abundant in muscle progenitor cells: insulin-like growth factor 1 (IGF1), an important factor in muscle development and growth, and angiopoietin 1 (ANGPT1), a factor that regulates stem cell quiescence [118–120]. It seems that postnatal myofiber growth and regeneration is influenced by SC quiescence through ANGPT1 and differentiation and growth-promoting effects of IGF1, both produced by pericytes [120].

### Preclinical research with MABs

To test whether MABs were suitable for skeletal muscle regeneration and restoration of protein expression, the cells were transplanted in a variety of MD animal models. Morphological and functional recovery was seen in adult immunocompetent *α-SG null* mice, an animal model for LGMD, after i.a. delivery of MABs transduced with a lentiviral vector expressing α-sarcoglycan (SG). After three consecutive injections, embryonic vessel-derived MABs restored α-SG expression in >20% of the muscle fibers in four different muscles. Furthermore, a decrease in fibrosis and injured muscle fibers allowed treated mice to run three times longer as untreated mice on a rotarod [121].

Delivery was optimized by exposure of MABs to combined pretreatment with SDF-1 or TNFα and expression of α4-integrin [122]. This led to α-SG expression in >90% of muscle fibers in the tibialis anterior muscle of *α-SG-null* mice four months after only one i.a. injection. It restored protein expression to roughly 60% of wild-type levels.

After this promising result, the possibility to use an allogenic donor therapeutic cell pool was assessed. H2-mismatched MABs from *BalbC* mice were transplanted in the same dystrophic mice. To assess the immune response against the donor cells, only half of the *α-SG-null* mice were treated with different immunosuppression. Under immune suppressive regime, donor cells formed more α-SG expressing fibers then did syngeneic MABs [123]. This observation indicates that with the correct immune modulation transplantation of MABs into an immunologically unrelated host can lead to long-term survival of donor cells that are able to form α-SG expressing fibers.

Finally, the formation of *de novo* muscle fibers was established when MABs derived from juvenile *C57Bl/6* mice were i.m. injected into dystrophin- and utrophin-deficient double knockout (*mdx|utrn-/-*) mice, a phenotypic model for DMD. Dystrophin expression was restored to approximately 50%, as compared to wild-type control mice, in the injected gastrocnemius [124].

### Preclinical research using pericytes

MABs seem perfect for the regeneration of dystrophic muscle, but with one important limitation: their isolation from the human aorta is challenging for clinical translation. Fortunately, pericytes can be more easily obtained by a skeletal muscle biopsy from MD patients or HLA-matched donors. The occurrence of pericytes in all vascularized postnatal organs makes them an attractive cell population [125]. It should be noted that cells isolated from other sources such as bone marrow, the atria and ventricles of the heart, and skeletal muscle are sometimes confusingly referred to as MAB- or pericyte-derived cells [126].

Pericytes performed comparable to MABs and restored *α-SG* expression in the *α-SG*-null mouse and produced dystrophin in the *scid/mdx* mouse after i.m. and i.a. injection. Both i.m. and i.a. injected mice showed enhanced functional performance after treatment, with treated mice running 50-80% more than untreated animals [127].

Sampaolesi and colleagues used progenitor cells obtained from muscle biopsies of Golden Retriever dogs as cell therapy in the GRMD model [128]. Ten dystrophic dogs were treated via local injections in the femur or systemic injections under different immune suppression regimes with steroids. Four dogs received autologous cells, transduced *in vitro* with a lentiviral vector expressing human microdystrophin. Six dogs were injected with wild-type progenitor cells from a leukocyte antigen-unrelated donor under treatment with either cyclosporine or rapamycin. The donor wild-type cells were more effective in alleviating dystrophy symptoms than the autologous, genetically corrected cells. Variable dystrophin expression was observed from different biopsy samples (10-70%) for all dogs and a functional increase in performance in the treated leg was seen. It was concluded that there was “remarkable clinical amelioration and preservation of active motility”. The notion that four of six dogs treated with donor cells showed an impressive clinical amelioration indeed calls for optimism and stimulates further research.

By the end of 2015, the first clinical study was published, revealing the safety of i.a. injection of HLA-matched donor cells in humans [129]. This exploratory, non-randomized open-label Phase I-IIa clinical trial was performed in five Duchenne patients. Variable doses of donor-derived cells were administered in limb arteries under immunosuppressive treatment four times at two-month intervals. One patient had a thalamic stroke without clinical ramifications, which was classified as unrelated to the intervention. Since safety was the primary outcome of this study, one could label it a success. Functional measurements showed stabilization but no functional improvement in two out of three ambulant patients. However, MRI showed disease progression in four of five patients. A follow-up study with inclusion of younger patients is needed to approach efficacy.

## Conditions and determinants of a successful muscle cell therapy

### Allo- or autotransplantation

**Fig. 2:**
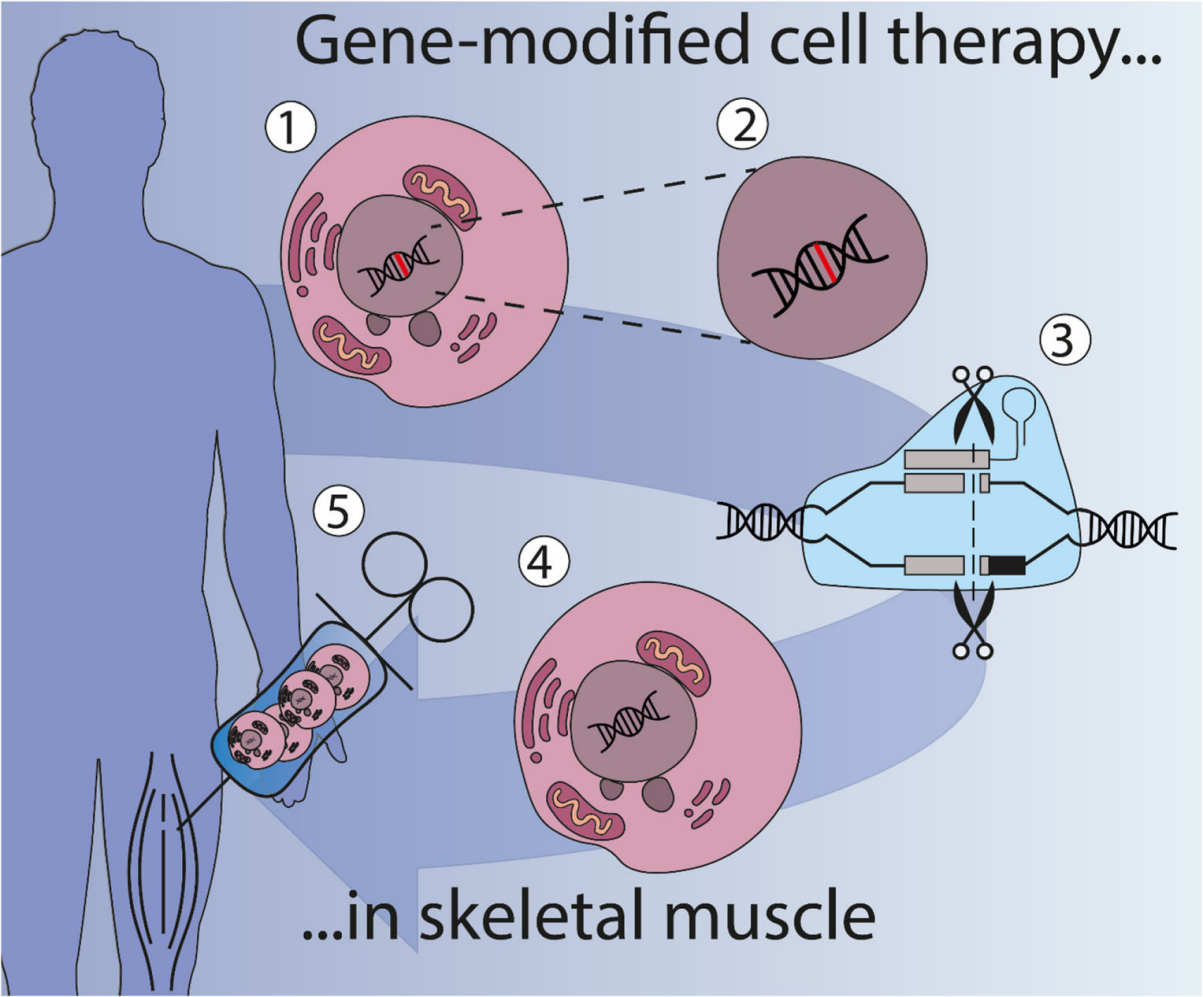
*Ex vivo* gene therapy in cells bridges cell and gene therapy. Cell therapy is the administration of cells into a patient with the goal of treating or curing a disease. One approach is gene-modified cell therapy, which is based on the isolation of cells from the patient (1) (autotransplantation), after which the mutated gene (in red) can be corrected (2) or a correct version can be introduced. Gene-editing technology like CRISPR/Cas9 is able to repair genes in the cell with high precision (3). Correctly edited cells (4) are then administered to the patient (5). There are no approved gene-editing treatments available in the clinic yet, but several are currently being researched in clinical trials (See clinicaltrials.gov)

Cell therapy for a genetic disease can be achieved with allotransplantation, where cells from a healthy donor are used, or with autotransplantation in which autologous cells from patients need to be genetically corrected first (Fig. 2) [4]. Allotransplantation requires chronic immunosuppression exposing patients to the risk of moderate to severe side effects. Immunosuppression can be avoided for an autograft transplantation, provided a reintroduced protein or vehicle used for genetic editing will not become immunogenic.

For some conditions the use of non-modified autologous cells is an option. OPMD, for example, is a late-onset progressive and autosomal dominant genetic disorder in which initially only a few distinct muscles are affected. The defects observed in myogenic cells isolated from affected muscles of patients with OPMD are not present in cell cultures originating from their unaffected muscles [19, 130]. Here, autologous cells can be obtained and expanded from clinically spared muscles and could represent a new therapeutic approach.

In most cases cell therapy and gene therapy must go hand-in-hand. For instance, in DMD many different muscles are affected and dystrophin expression needs to be restored by widespread supplementation of cells that are able to support muscle growth [131]. The *ex vivo* strategy begins with isolating cells from the patient followed by correcting the mutation or restoring the reading frame and producing functional dystrophin protein. The precursor cells that contribute to tissue repair can be seen as vectors since these are deployed to deliver the functional gene. In a different disease example, myotonic dystrophy, an RNA gain-of-function disease with a multisystemic manifestation, removal or correction of the unstable repeat expansion that evokes the toxic RNA effect in muscle cells is the ultimate goal [5, 131]. *Ex vivo* corrected muscle progenitor cells will have to counterbalance the existing endogenous RNA toxicity and thus restore muscle regeneration capacity, preferably by repopulating the muscle stem cell pool *in vivo*.

### *Ex vivo* gene editing or gene augmentation

Many forms of MDs are monogenic disorders, which makes gene replacement a promising concept. Clustered regularly-interspaced short palindromic repeats (CRISPR)/CRISPR-associated system (Cas) is a powerful tool to accurately alter a target in the genome of eukaryotes [132]. However, unwanted off-target cleavage events by CRISPR/Cas remain a bottleneck and especially unintended germline modifications are worrisome [133, 134].

In an *in vivo* approach, it is impossible to select for the correctly edited cells and thus the accuracy, efficiency and safety of the gene editing process needs to be exceptionally high. *Ex vivo* gene editing comes with the considerable benefit of selection of correctly edited cells. A drawback of this approach, however, can be the size of the correctly edited cell pool that is needed. Freshly isolated myogenic precursor cells have the highest regenerative potential and culture conditions influence transplantation efficiency in a negative manner [135, 136]. The prolonged culture to amplify the few correctly edited cells will likely also lead to a less effective cell pool.

### *In vitro* expansion of muscle precursor cells while maintaining therapeutic potential

The clinical trials included in this review (Table 3) were mostly focused on safety and feasibility [59, 78]. When functional effects are envisioned, larger cell doses are needed. Obtaining more cells is challenging since *in vitro* expansion under traditional culture conditions is often hampered by changes in phenotypic expression of cells, which in turn affects transplantation efficiency.

Cells in an artificial *in vitro* environment are exposed to non-physiological conditions such as the non-elastic plastic culture substrate, high oxygen supply, additional supplements and loss of contact with other cells. These circumstances affect cell behavior at that moment, but also have long-lasting effects on cells after transplantation. It is well established that expansion of myogenic cells before engraftment reduces their regenerative capacity [136, 137]. This is probably caused by a more activated and differentiated state of the grafted cells, which may diminish their regenerative potential, since freshly isolated progenitor cells are not activated at the time of grafting [136, 137].

One way to reduce culture-induced modifications is via the use of soft hydrogel substrates to mimic tissue rigidity, a biophysical property of the skeletal muscle microenvironment [138, 139]. The altered substrate rigidity is thought to preserve stemness by altering cell shape. Mechanical sensing signals are transmitted via focal adhesions towards nonmuscle myosin II isoforms (NMM IIA, B, and C). These non-muscle myosins signal cells to deform their matrix which results in cytoskeletal rearrangements and altered signaling [140, 141] [142, 143]. MSCs, for example, express no baseline levels of lineage-specific markers however the substrate rigidness influences lineage specification of these naive stem cells. Softer matrices of polyacrylamide coated with collagen I that mimic brain tissue are neurogenic for MSCs, while similar but stiffer matrices induce myogenic differentiation and rigid matrices give rise to osteogenic cells [140]. When cells are in identical serum conditions, the matrix stiffness is a stronger driving force for reprogramming MSCs than are soluble factors. However, the combination of correct elasticity and soluble induction factors synergistically cause an even more complete myogenesis [140].

Typically used polystyrene plastic has an elastic modus of ~3 GPa which is five orders of magnitude more rigid than skeletal muscle [138, 144]. Using tunable polyethylene glycol (PEG) hydrogel and laminin as an adhesion ligand, MuSC division rate remained unchanged and MuSC stemness was retained, as seen in the gene expression [138]. Hydrogels are effective in keeping the proliferative capacity stable in expansion cultures up to 20 passages *in vitro* [138, 139]. However the extent of engraftment from cultures MuSC is still not as high as that of freshly isolated cells, suggesting that additional chemical and physical cues may be required to establish an optimal *in vitro* muscle microenvironment to keep the maximal therapeutic potency [138].

Besides tissue rigidity, specific extracellular matrix (ECM) protein coatings can be used to mimic the SC niche. Matrigel, the most widely used commercialized ECM mixture, albeit without FDA approval, contains critical grow factors and cytokines [145, 146]. The most prevalent proteins in the mixture are laminins, collagens I and IV, and fibronectin. These proteins are also present in the ECM of the myofiber niche [147]. Laminin mainly affects proliferation and migration of myogenic progenitor cells, while fibronectin plays a role in remodeling after muscle damage [148]. Under culture conditions fibronectin can modulate cellular expansion by potentiating the Wnt7a-dependent signaling pathway [149]. Collagen is more of a structural component, it improved the self-renewal of SC [147, 150]. Several peptides released from ECM proteins called matricryptins have been suggested to be useful in the proliferation, migration, and survival rates of myoblasts [151, 152] although specific research is missing.

Other than mimicking the *in vivo* environment to aid cell expansion, proliferation of cells can be influenced by the use of nitric oxide, calorie mimicking drugs, or genetic modification of specific targets. Nitric oxide (NO) contributes to myogenesis by the formation of S-nitrosothiols (RSNO). An increase in RSNO inhibits the S-nitrosoglutathione reductase (GSNOR) pathway and leads to an increase in the number of myoblasts, followed by a decrease in the myoblast fusion index [153].

Myoblasts cultured in the presence of calorie restriction mimetics behave differently. These drugs, for example metformin and ursolic acid, regulate myogenic differentiation and cell proliferation depending on the dose. By varying the doses of the calorie restriction mimicking drugs one can induce a shift between proliferating and differentiating status [151, 154, 155]. Genetic modification of cells is also advocated to promote expansion, engraftment and sustained regeneration. Transcriptome studies revealed that activation of p38 signaling correlated with myogenic cell differentiation, while inhibition of p38 reversibly prevented differentiation and could be used *ex vivo* to promote expansion. Pharmacologic manipulation of p38 signaling can be leveraged for enhancement of both *ex vivo* expansion and subsequent *in vivo* engraftment of cells [135].

### The value of using iPSCs in muscle cell therapy

A different approach to circumvent the limited availability of freshly isolated primary cells, low proliferation capacity and early senescence, is the use of induced pluripotent stem cells (iPSCs). Generation of iPSCs is achieved by reprogramming somatic cells with a defined set of transcription factors (*OCT3/4*, *SOX2*, *c-MYC*, *KLF4*) [156]. These so-called Yamanaka factors can be supplied by integrating and non-integrating viral vectors or non-viral episomal vectors to mitigate the risk for insertional mutagenesis. Next to their unlimited replicative capacity, another advantage of the use of iPSCs is the possibility to select correctly edited cells after CRISPR/Cas treatment. These can then be clonally expanded and differentiated into suitable muscle progenitor cells [157].

Genetically corrected human iPSC-derived MAB-like cells (HIDEMs) from LGMD patient fibroblasts only restored α-SG in 2% of skeletal myofibers in *α-SG-KO* mice after i.m. injection [117]. The relatively low engraftment of donor cells suggests that autologous therapy using genetically corrected HIDEMs might be difficult. Efficiency of transplantation of these cells seems to be low, indicating a delicate balance between proliferative capacity and differentiation potential for the ultimate transplantable cell [117].

As mentioned before, progeny-specific imprinting plays a role in differentiation [24, 25]. Stronger myogenic commitment can be achieved via the generation of iPSCs from prospectively sorted myogenic cells (here called MAB-iPSCs) instead of fibroblast-derived iPSCs (f-iPSCs). The epigenetic memory in MAB-iPSCs provides a more robust myogenic differentiation [25]. I.m. injection of wild-type mouse iPSC-derived MAB-like cells (MIDEMs) resulted in an amelioration of the motor capacity in *Sgca-null/scid/beige* mice [158]. Although not compared one-on-one, efficacy of (genetically corrected autologous) HIDEMs is likely higher compared to f-iPSCs *in vivo*.

### Survival and engraftment of transplanted cells *in vivo*

A crucial factor for an effective cell therapy is the early survival of donor cells. The ultimate goal is that the ratio between death and proliferation leads to a good “net” survival post-transplantation, with the whole donor cell population being either stable or growing. Although suggested many times, it seems that myogenic cells are not instantly killed and removed after i.m. injection, but they undergo necrosis or apoptosis in the first few days. Factors that have been implicated in cell death are cellular effectors of an acute inflammatory response, a combination of natural killer cells, T-lymphocytes and complement, and the fact that only a small specific donor cell sub-population with stem cell-like characteristics survives [159].

Various factors are able to improve the survival, migration and engraftment of injected cells. The growth factor, vascular endothelial growth factor (VEGF) reduced hypoxia-induced death of human myoblasts *in vitro* and in a mouse model [151, 160]. Other growth factors such as insulin-like growth factor-1 (IGF-1) and basic fibroblast growth factor (bFGF) were co-injected and promoted the overall migration of human myoblasts in various mouse models and stimulated cell migration and engraftment of monkey myoblasts in a nonhuman primate model [161]. Treatments with these factors stimulated components of proteolytic systems and thereby enhanced cellular migration. A short term *ex vivo* treatment using Wnt7a, a member of the Wingless-INT (WNT) family, on satellite stem cells had similar effects and markedly increased cell dispersion and engraftment, which ultimately resulted in improved muscle function of dystrophic muscles [162].

The inhibition of p38 signaling pathway *in vitro* during SC expansion enhances *in vivo* engraftment [135]. Identifying additional modifications in the culture protocol that increase the regenerative potential of isolated cells will therefore have large beneficial effects. As an example, improvements have been made by p53 inhibition which can increase survival of edited cells. Nonetheless, the selection of p53-mutated cells raises additional concerns, since p53 inhibition could increase cancer risks [163, 164].

### Pre-treatment of receiving skeletal muscle

The harsh environment that cells get exposed to after injection likely plays a role in rapid cell death and low long term viability [165]. MDs cause changes in the skeletal muscle niche leading to a hostile environment for injected cells [166, 167]. The use of immunosuppressive drugs has proven to increase viability of injected cells however it comes with side effects [168]. The encapsulation of cells in polymer-based microcapsules might be effective. The encapsulated cells are protected from the immune system but outward diffusion of factors is still possible [169]. The review of Murua *et al.* focusses on the cell microencapsulation technology and the possibilities in the clinic [169].

Fibrosis, the progressive replacement of functional tissue by nonfunctional and more rigid connective tissue, occurs in many MDs. In a fibrotic environment cells have been shown to differentiate more towards a fibrogenic fate thereby decreasing their regenerative potential [170, 171]. Improving the quality of receiver muscle by antifibrotic therapies before grafting will be helpful to optimize the fate of implanted cells. The co-injection of myoblasts with other cell types such as pro-inflammatory macrophages increased proliferation and migration, and delayed differentiation [172].

As mentioned before, alterations in Wnt7a and p38 signaling could exert positive effects on myogenic cell migration and engraftment. Besides pretreatment of cells, treating receiving muscle tissues could also potentially be beneficial [135, 173]. Focal treatment of dystrophic muscles using *mdx* mice showed that Wnt7a treatment efficiently induced satellite cell expansion, myofiber hypertrophy, reduced the level of contractile damage and caused a shift in fiber type toward slow-twitch [174].

### The competitive potential of transplanted cells

A crucial factor complicating the efficacy of myogenic cell transplantation is that both mRNA and intracellular proteins in myofibers tend to stay close to the nucleus of origin, in an area called the nuclear domain [175]. Due to this limited spreading, proteins from donor cells remain in the myofiber to which the transplanted cells fused. The size of the nuclear domain differs for each protein, but also depends on the length of the myofiber in which grafted cells are integrated. For DMD, the graft-derived myonuclei can transcribe mRNA and synthesize donor cell-derived dystrophin. In a handful of clinical trials with myoblast transfer, expression of donor-derived dystrophin was indeed confirmed in myofibers of patients [30, 46, 47, 176]. However, the novel proteins remained near the donor myonucleus, the nuclear domain in the myofiber. Muscle sections derived from DMD patients that underwent cell engraftment with healthy myoblasts showed that donor-derived dystrophin expression was restricted to distances of 0.7-2.0 mm in single myofibers [47].

Importantly, in myotonic dystrophy there is no need to ensure distribution of a missing protein along a myofiber, but the removal or reduction of the toxic RNA in a myotube is required for functional improvement. Therefore, once the technical procedures for *ex vivo* editing and cell transplantation have been established, further studies are required to determine conditions of the most optimal administration and dosage regimen to ensure the regeneration of myofibers and exert a positive effect on muscle function.

### Timing the intervention

For most patients, the diagnosis of MD comes after the onset of symptoms. An important question therefore is whether a proposed treatment can still be effective at or after this time point. Secondary pathologies that arise, due to the genetic defect, are known to cause irreversible damage. For example, DMD boys are generally diagnosed at the age of 4-5 years. Muscle biopsies at that time point already show hallmarks of the dystrophic progress. In general, various forms of MD show progressive replacement of muscle tissue by connective and adipose tissue, rendering muscle increasingly weak and nonfunctional [177].

A cell therapy differs from a gene therapy approach in that it not only corrects a gene, but also delivers a pool of healthy myogenic cells for the regeneration process. Consequently, gene therapy might not be effective at later disease stages, while cell transplantation might still halt disease progression. An illustration of this idea is provided in a study were a BMD patient with progressive symptoms for eleven years underwent autologous bone marrow mononuclear cell transplantation and multidisciplinary rehabilitation for nine months. Gradual improvement in muscle strength and respiratory function indicated halting of disease progression [178].

Studies examining the age dependence of cell therapy for MDs are missing. Gene transfer studies that investigate the age-dependent effect on treatment efficiency for other genetic disorders have demonstrated that earlier treatment increases the therapeutic effects [179]. If we assume that clinical experience will mirror these preclinical findings, the advancement and quick implementation of improved diagnostic procedures is of utmost importance.

The best way to identify patients ahead of symptom manifestation, when a treatment will be most effective, is with carrier testing in families with a history of MDs and newborn screening for *de novo* mutations [179]. DMD has been included (often as a pilot study) in newborn screening programs in Edinburgh (UK), Germany, Canada, France, Wales, Cyprus, Belgium, Australia, China and the USA [180]. A heel prick, taken shortly after birth, provides a bloodspot in which serum creatine kinase (CK) elevation can be established in DMD cases [181]. Nowadays, there is no universal newborn screening for any of the MDs, however this may change soon. Many public health organizations around the world attempted to screen for, or are planning to screen for MDs.

## Summarizing remarks and outlook

The muscular dystrophies (MDs) are highly variable in terms of age of onset, severity of symptoms, clinical pattern and genetics. Despite enormous research efforts in the past decades, there is currently no cure for any (sub)type of MD. This may change in the future by implementing cell therapy, but before the use of stem cells for muscle regeneration can become clinical reality there are several challenges that need to be met.

To identify the ultimate muscle progenitor cell for cell therapy, we need more clarity among the cells currently at our disposal. A successful muscle progenitor can efficiently proliferate, survive *in vivo*, disperse throughout the muscles, repopulate muscle stem cell niches and fuse with existing fibers to efficiently differentiate into functional muscle. Established and validated cell isolation protocols, clear characterization and workable culture protocols are crucial, and additional research will lead us to the cell type that best fits the requirements for successful restoration of muscle morphology and function.

Nowadays, myoblasts and satellite cells are no longer considered to be suitable due to their prerequisite of local injection. However, in this review we have discussed additional and promising cell types such as ALDH^+^ cells, CD133^+^ cells, MSCs, MDSCs, MuStem cells, SP cells, myoendothelial cells, PICs and MABs and pericytes. Each with their own merits and inherent limitations. Some of these cell types show high myogenicity, but are difficult to expand *in vitro*, while others can be isolated and propagated in workable amounts, but show *in vivo* inefficient myogenic differentiation or are unable to negotiate the vessel wall when systemically delivered. At the current time, pericytes show highly promising results *in vitro* and crucially *in vivo*, in a mouse model of MD and more recently in the dystrophic dog, although the subsequent clinical trial was inconclusive. Besides, the discovery and expanding application of iPSCs has unlocked a new research area, from which we expect exciting findings. IPSCs also offer hope for fulfillment of the above criteria, especially when gene editing of autologous cells is needed to correct the underlying genetic defects that lead to MDs.

Although each MD is different and each (sub)type might need a slightly different strategy in terms of cell dose, administration, timing and editing strategy, research is progressing and clinical trials are activated thereby bringing hope that effective therapies are on the horizon.

## Data Availability

Not applicable.
